# Dr. Gordon Dill's Provident Scheme

**Published:** 1921-01-22

**Authors:** 


### HOSPITAL AND MEDICAL SERVICES FOR SUSSEX.

### Dr. Gordon Dill's Provident Scheme.

By the help and co-operation of the principal hospitals in Brighton, Hove, and Preston, a provident scheme has been established which will supply its members, free of cost, with practically all the resources of medicine which can be afforded locally. This scheme, the rules of which we give in full, has been organised by Dr. J. Gordon Dill, O.B.E. It has nothing whatever to do with the National Health Insurance or any other organisation for medical benefit, and it does not provide its members with ordinary medical (*i.e.*, general practitioner) attendance, or other benefit to which they are entitled from the State or from local authorities.

The scheme is for the benefit of those who, being *bona fide* residents of Sussex, irrespective of class or occupation, are in a financial position which makes them eligible for election as members. All those insured under the National Health Insurance Acts are eligible for membership, but while for others it is obviously impossible to fix a hard-and-fast line of financial eligibility, a maximum income limit of £260 for single members, £400 for married people without children, or for a widow or widower with one child, and £500 for a family may perhaps be assumed generally.

### The Facilities Provided.

The following are the facilities which have been arranged: The medical attendant of a member may arrange, by appointment, for private consultations at any of the co-operating hospitals, and such treatment as he and the consultant may jointly think advisable will be undertaken at the hospital if this should be necessary. For those members who are living in the County Borough of Brighton and Municipal Borough of Hove, and unable to leave their beds, the services of visiting consultants may be secured by appointment. For country members unable to leave their beds, free consultations may be obtained by appointment, subject to the payment to the consultant at the time of consultation of a charge equal to one-half of the usual mileage rates calculated from the Brighton Central Railway Station. The Brighton and Hove Provident Dental Hospital will supply members sent by their medical attendant with all ordinary dental treatment which may be considered necessary, and will provide them with any dentures which may be required at hospital rates.

### Additional Medical Services.

In addition to consultations and dental treatment, the following services are available for the benefit of members, after consultation, in suitable cases: All the resources of the Stephen Ralli Memorial Laboratory, Royal Sussex County Hospital, including bacteriological and pathological investigations and examinations beyond the province of general practice; *x*-ray examinations at the Royal Sussex County Hospital and at the Western Branch of the Brighton, Hove, and Preston Dispensary, at the request of the medical attendant. Treatment by *x*-rays (including bismuth meals with examinations) will be given, and massage and electrical treatment at the Royal Sussex County and other Hospitals.

### Hospital Treatment.

Urgent cases are admitted to hospitals as at present. Other cases requiring operation or other hospital treatment will, after consultation, be admitted to hospital, or be placed upon the waiting-list of the hospital until there is a vacant bed, but a member is not to take precedence over more urgent cases, and the ordinary hospital routine is not to be disturbed. In all the co-operating hospitals in which beds are reserved at present for paying patients, members will be admitted to these beds if available, should they desire it, at a uniform reduction of one guinea a week from the usual price of the bed.

### The Co-operating Hospitals.

The Royal Sussex County Hospital, the Royal Alexandra Hospital for Sick Children, the Brighton and Hove Hospital for Women (for obstetrics and gynæcology), the Brighton and Hove Provident Dental Hospital (for dental surgery), the Brighton, Hove, and Preston Dispensary, the Sussex Eye Hospital (for ophthalmic cases), the Sussex Throat and Ear Hospital (for diseases of the throat, nose, and ear), the Lady Chichester Hospital (for borderland mental cases), and the New Sussex Hospital for Women and Children are co-operating in this scheme.

### Rules.

1. No one will be accepted as a single member except an unmarried man or woman, or a widower or widow without children under the age of sixteen. The subscription for a single member will be £1 per annum.

2. Married people without children, or a widower or widow with only one child under the age of sixteen years, may subscribe jointly at a rate of £1 10s. per annum for the two, although each shall be accounted a member.

3. Married people with a child or children under the age of sixteen, or a widower or widow with children under the age of sixteen years, may only subscribe at a family rate of £2 per annum, which shall include the whole family, however large, except children over the age of sixteen, who must be accepted as single members, although each individual in the family shall be accounted a member. N.B.—These rates are subject to revision by the co-operating hospitals after due notice.

4. All candidates for membership must be approved by the "Committee of Reference" formed by the co-operating hospitals, except in the case of those insured under the National Health Insurance Acts, who will produce their medical card. In the event of the eligibility of any member being questioned, the matter will be submitted to the "Committee of Reference," whose decision shall be final.

5. Cards of membership, bearing the date, will be issued on the first day of each month to those who have become members during the preceding month, and the benefits will begin on the date of the card, and continue for one calendar year. These cards can be produced as evidence of membership.

6. At the end of one year from the date of issue of the membership card the benefits will cease unless the subscription has been renewed. Fourteen days' grace will be given for renewal (during which no benefit will be available), but, after this, renewed membership will only be possible from the beginning of the next quarter.

7. When the number of members has reached the limit of the capacity of the co-operative hospitals the list will be closed

8. When a member is leaving the county within the first six months of membership, and in consequence wishes to resign, one-half of the subscription will be repayable.

The first ten thousand members receive an "original member's" card, and their subscription will not be raised.

